# Work-related burnout among public secondary school teachers is significantly influenced by the psychosocial work factors: a cross-sectional study from Ethiopia

**DOI:** 10.3389/fpsyg.2023.1215421

**Published:** 2023-07-03

**Authors:** Azanaw Asega Belay, Kassahun Ayele Gasheya, Garedew Tadege Engdaw, Gebisa Guyasa Kabito, Amensisa Hailu Tesfaye

**Affiliations:** ^1^Department of Occupational Health and Safety, College of Medicine and Health Sciences, Wollo University, Dessie, Ethiopia; ^2^Department of Environmental and Occupational Health and Safety, College of Medicine and Health Sciences, Institute of Public Health, University of Gondar, Gondar, Ethiopia

**Keywords:** Copenhagen Burnout Inventory, work-related burnout, school teachers, Ethiopia, psychosocial work factors

## Abstract

**Introduction:**

Work-related burnout (WRB) is the degree of physical and psychological fatigue and exhaustion perceived by individuals as related to their work. Even though the condition is widespread across various occupations, teachers inevitably experience high levels of burnout in their work, which can have long-term effects on their health and well-being. However, in developing countries such as Ethiopia, the lack of reliable data on psychosocial hazards, including work-related burnout, often encumbers officials from planning preventive measures. This study investigated the prevalence and contributing factors of work-related burnout among public secondary school teachers in Gondar City, northwestern Ethiopia.

**Methods:**

An institution-based cross-sectional study was conducted from May to June 2022. A sample of 588 teachers was recruited using simple random sampling. Work-related burnout was assessed using a standardized seven-item Copenhagen Burnout Inventory (CBI). The data were collected through a self-administered questionnaire. The collected data were entered into EpiData and analyzed using SPSS. A multivariable logistic regression analysis was used to identify factors associated with work-related burnout.

**Results:**

The overall response rate was 94.05% (*N* = 553). The majority of participants, 356 (64.4%), were male. The mean (±SD) age of the participants was 38.74 (±7.65) years. This study demonstrated that the prevalence of work-related burnout among school teachers in the past 12 months was 37.4% (*n* = 207). High job demands, job stress, job dissatisfaction, low role clarity, and student demotivation were found to be the psychosocial work factors that significantly influenced the prevalence of work-related burnout among school teachers.

**Conclusion:**

This study highlights the high prevalence of work-related burnout among public secondary school teachers. Psychosocial work factors such as job demands, job stress, job satisfaction, role clarity, and student demotivation were significant factors influencing work-related burnout. To reduce the condition, it is recommended to take measures to cope with high job demands, improve stress management skills, promote job satisfaction strategies, clarify teachers’ responsibilities, and use effective teaching practices to motivate students. In general, addressing psychosocial work factors needs to be central to efforts to prevent teacher burnout.

## Introduction

Burnout is a state of physical, mental, and emotional exhaustion (Piperac et al., 2021). Burnout occurs as a result of sustained stress that does not abate (Maslach and Leiter, 2016). This is due to prolonged periods of intensity and excessive demands on energy, strength and resources (Ahola, 2007). The World Health Organization (WHO) has classified burnout as an “occupational phenomenon” in the 11^th^ revision of the International Classification of Diseases (ICD-11) (World Health Organization, 2020). Work-related burnout (WRB) is the degree of physical and psychological fatigue and exhaustion perceived by the individual as related to their work (Bauer et al., 2006). Work-related burnout is a psychological syndrome that results from the ineffective management of prolonged work-related stressors (World Health Organization, 2019). It is a growing and widespread global health problem, affecting a significant proportion of the working population in every country (Kerry-Henkel, 2017; Mogapi and Moorad, 2020; Montoya et al., 2021).

Education is critical to achieving the third goal of the Sustainable Development Goals (SDGs), which relates to health and well-being. As highlighted by the United Nations Educational, Scientific and Cultural Organization (Saini et al., 2023), education is key to informing people about drug and alcohol abuse and prevention, as well as mental health issues. It also provides relevant knowledge and information on family planning, sex education, and reproductive health. Teachers are responsible for preparing future generations to face the challenges of current unsustainable development (García-González et al., 2020). Therefore, teachers need to preserve and maintain their psychological well-being to face the various challenges in the education sector.

In addition to teaching, teachers today have a heavy daily burden of administrative tasks such as documentation and program implementation (Ali et al., 2017; Musa et al., 2018). Teaching, in particular, involves long hours, and a lot of work, and is emotionally demanding for the government, students, and parents (Tang et al., 2013). A typical teacher’s day starts early in the morning and consists of about five lessons, including extracurricular activities and other teaching responsibilities such as assessments and reports. Because many teachers are judged on the objective results that grades can provide for evaluation purposes, marking takes up a significant amount of non-teaching time (Morris, 1999). Teachers have begun to express concerns about their ability to control their students. Although teachers inevitably experience high levels of burnout in their work, this can have long-term effects on their health and well-being (Tang et al., 2001; Lizano, 2015). Work-related burnout (WRB) is a major predictor of high absenteeism, turnover, poor interpersonal relationships, and increased accidents among school teachers (Mogapi and Moorad, 2020).

The prevalence of WRB among teachers varies between countries. Work-related burnout among public school teachers has been reported to be 47% in Germany (Seibt and Kreuzfeld, 2021), 52% in the United States (Rumschlag, 2017), 73.3% in Indonesia (Ramdan et al., 2020), 19.5% in Italy (Pellerone et al., 2020), 50% in Malaysia (Subon and Sigie, 2016), 36% in Nigeria (Ozoemena et al., 2021), 28.8% in Namibia (Louw et al., 2011), 40% in Botswana (Mogapi and Moorad, 2020), 27.5% in Tunisia (Chennoufi et al., 2012), and 54.2% in Ethiopia (Wulolign et al., 2020), indicating a significant public health problem.

Several factors contribute to WRB among secondary school teachers (Arvidsson et al., 2016; Kepekepe et al., 2017; Carlotto and Câmara, 2019; Jamaludin and You, 2019; Posada-Quintero et al., 2020; Shaheen and Mahmood, 2020). There is evidence that the risk of experiencing burnout is associated with gender, years of teaching experience and the grades taught (Brewer and Shapard, 2004; Pyhältö et al., 2021). For example, female teachers are more likely to experience higher levels of work stress and exhaustion than male teachers, who in turn are more likely to suffer from cynicism (Skaalvik and Skaalvik, 2017). In addition, early career teachers appear to be more vulnerable to burnout than more experienced teachers (Brewer and Shapard, 2004). Teaching in secondary schools is very difficult and emotionally draining as teachers deal with a wide range of teenagers who are challenging and misbehaving (Marić et al., 2020). While for many decades teaching was one of the most respected professions in Ethiopia, it has now lost its status, and secondary school teachers are treated with minimal respect and status by the community and students (Mengistu, 2012). Teacher burnout is associated with classroom management, including emotional climate, dyadic teacher-student relationships, and the interpersonal conflicts with students, parents or peers (McCarthy et al., 2016). Researchers have investigated that teacher burnout results from an imbalance between teaching demands (e.g., problematic student behavior, administrative demands) and teaching resources (e.g., school support staff, availability of teaching materials) (Iancu et al., 2018). In addition, it is an observable condition that students in secondary schools show demotivation and lack of interest in learning and studying as a result of the political crisis, and the economic and educational situation in Ethiopia (Jones et al., 2022), which in turn affects the psychological condition of school teachers, leading to burnout (Sharifian and Kennedy, 2019). The psychological health factors associated with WRB have been investigated in several studies. These factors included distress, depression, anxiety, suicidal ideation, personality disorders, and personality traits (Meredith et al., 2022).

Workplace burnout needs to be addressed immediately as it is the most significant health issue affecting the current delivery of education services in Ethiopia. However, there is little evidence on the extent of WRB and its associated factors among public secondary school teachers. Therefore, the present study was conducted to investigate the prevalence and factors associated with work-related burnout among public secondary school teachers in Gondar City, Ethiopia. The results of this study could provide educational leaders with baseline information to design and promote wellness programs for targeted professionals to minimize and control work-related burnout. It will also provide relevant data for policymakers and stakeholders to design and implement effective prevention and control strategies to reduce further incidences.

## Materials and methods

### Study design, period, and setting

An institution-based cross-sectional study was conducted from 30 May to 22 June 2022. The study was conducted among teachers in public secondary schools in Gondar City. Gondar City is the oldest and most historic city in northwestern Ethiopia. It is located about 727 km from Addis Ababa, the capital of Ethiopia, and 180 km from Bahir Dar, the capital of the Amhara Regional State. There was a total of 12 public schools and about 838 public secondary school teachers in Gondar city at the time of the data collection period.

### Populations and eligibility criteria

The study population consisted of all public secondary school teachers in Gondar City. Public secondary school teachers who had worked for at least 12 months before the study were eligible for this study (Wulolign et al., 2020), while those on annual leave, maternity leave, and sick leave during the data collection period were excluded.

### Sample size determination and sampling procedure

The sample size was calculated using a single population proportion formula (Charan and Biswas, 2013) with the following assumptions: 4% margin of error (d), proportion (p) of work-related burnout among teachers 33.4%, which was obtained from a previous study (Wulolign et al., 2020), and Zα/2 = the value of the standard normal curve score corresponding to the given confidence interval. Accordingly, based on a single population proportion formula: $n={\left(Z\alpha {/}_{2}\right)}^{2}\;\frac{\left[p\;\left(1-p\right)\right]}{d^{2}}$ ; where *n* = initial sample size, Zα/2 = 1.96 corresponding to 95% confidence level, p = proportion = 33.4%, d = margin of error = 4%; $n={\left(1.96\right)}^{2}\;\frac{\left[0.334\;\left(1-0.334\right)\right]}{0.04^{2}}$ =534. The total sample size for this study was 588 after adding a 10% contingency for the non-response rate.

A simple random sampling technique was used to recruit eligible samples. A sample was drawn from each of the public secondary schools in Gondar City. To select the participants in each school, the calculated sample size was proportionally distributed across all 12 public secondary schools in Gondar City. The required sample size from each school was then randomly selected using computer-generated random numbers, using the list of teachers obtained from the Human Resources Department as the sampling frame.

### Operational definitions and measurement of variables

#### Work-related burnout

Work-related burnout was measured using the seven-item Copenhagen Burnout Inventory (CBI) questionnaire (Kristensen et al., 2005), and the presence or absence of WRB was determined based on the average total score. A total score of 50 or higher on the CBI was classified as the presence of WRB, whereas a score below 50 was classified as the absence of WRB (Borritz and Kristensen, 2004).

#### Secondary school teachers

Teachers who are assigned to teach grades 9 to 12.

#### Body mass index

weight in kilograms divided by the square of the height in meters (kg/m^2^), categorized as underweight = BMI < 18.5, normal = BMI 18.5–24.9, overweight = BMI ≥ 25 (Seidell and Flegal, 1997).

#### Doing physical exercise

Exercising or doing any kind of sports activity at least two times per week with a duration of at least 30 min (Rolander and Bellner, 2001; Tesfaye et al., 2021).

#### Alcohol drinking

The consumption of any type of alcoholic-based beverage, whether locally or industrially produced, by teachers in any quantity at least twice per week (Nakata et al., 2006).

#### Khat chewing

Chewing khat in any form or volume three times a week for at least 12 months (Melchior et al., 2003; Gebremichael and Kumie, 2015). Khat is a plant whose leaves are chewed or dried, crushed, and drunk as tea in the East African region, as well as some parts of the Middle East, for stimulation or enlightenment (Lemessa, 2001; Ben-Shabat et al., 2014).

#### Cigarette smoking

It is the practice of smoking a cigarette by teachers for at least one stick of cigarette per day (Nakata et al., 2006).

#### Psychosocial work factors

These include psychosocial dimensions such as job stress, depressive symptoms, job satisfaction, role clarity, job demand, sleep trouble, social support, social relation and are measured by the Copenhagen Psychosocial Questionnaire (COPSOQ). The COPSOQ is an international instrument designed to assess psychosocial conditions in any type of workplace. Each psychosocial dimension contains different items with possible responses on a five-point Likert scale and dichotomized on the basis of their mean scores (Kristensen et al., 2000; Henny et al., 2014; Llorens et al., 2019).

#### Student misbehavior

Student misbehavior was measured by 3 items related to student discipline (5-point Likert scale ranging from strongly disagree to strongly agree, *α* = 0.83). A score of 15 or higher indicates that participants are experiencing problems due to student misbehavior (Aldrup et al., 2018).

#### Student demotivation

Student demotivation was measured by 4 items related to school work (5-point Likert scale ranging from strongly disagree to strongly agree, *α* = 0.85). A score of 19 or higher indicates that participants experience problems due to student demotivation (Amri et al., 2021).

### Data collection tools and procedure

Data were collected using a standardized self-administered Copenhagen Burnout Inventory (CBI) questionnaire (Kristensen et al., 2005) after the investigators reviewed and modified various relevant literature based on the study objectives (Asrat et al., 2015; Biksegn et al., 2016). The questionnaire was originally written in English and then translated into Amharic, the local language of the study area, by the authors and language experts to ensure that respondents could understand the questionnaire and to ensure consistency with the corresponding English version. The survey questions comprise four sections. The first section includes socio-demographic factors such as age, sex, level of education, teaching experience, household members in person, and monthly salary. The second part of the questionnaire includes behavioral factors such as body mass index (kg/m^2^), physical activity (yes/no), cigarette smoking (yes/no), alcohol consumption (yes/no), and khat chewing (yes/no).

The third section of the questionnaire focused on gathering information about teachers’ work-related burnout and was collected using the CBI questionnaire. The CBI questionnaire consists of three sub-dimensions measuring personal burnout, work-related burnout (WRB), and client-related burnout. The three separate parts of the questionnaire were designed to be used in different settings. However, in this study, we used the WRB domains of the CBI. Work-related burnout was defined as “the degree of physical and psychological fatigue and exhaustion that the person perceives as related to his or her work (Kristensen et al., 2005).” The work-related burnout domain of the CBI questionnaire consisted of seven items consisting of a five-point Likert scale ranging from 1 to 5 with a scoring system of 0 to 100. Responses to each item ranged from very low/never = 1, low/rarely = 2, somewhat/sometimes = 3, high/often = 4, and to very high/always = 5. Following the instructions of the questionnaire designers, the scale labels were recoded to the format 1 = 0, 2 = 25, 3 = 50, 4 = 75, and 5 = 100. Instead of having some ‘positive’ and some ‘negative’ items, we decided to key all items (except one) in the same direction. Accordingly, we reversed the scores on a positive item, e.g., 1 = 5, 2 = 4, 3 = 3, etc., and then summed all seven items (Kristensen et al., 2005; Creedy et al., 2017; Piperac et al., 2021). Based on the tool’s scoring system, we summed all scores out of 100 and dichotomized a score of <50 = 0 (work-related burnout) and a score of ≥50 = 1 (work-related burnout) (Borritz and Kristensen, 2004). The CBI questionnaire has been validated in different countries and populations and has been found to have very high internal reliability (Fiorilli et al., 2015; Mahmoudi et al., 2017; Papaefstathiou et al., 2019; Rocha et al., 2020; Wood et al., 2020). For instance, the CBI validation among Nigerian resident doctors yielded a Cronbach alpha (α) score of 0.87. It has also been translated into several languages and is currently used in many countries (Kristensen et al., 2005; Ogunsuji et al., 2022). In this study, Cronbach’s alpha for the WRB domain (7-items) of the CBI was found to be reliable, Cronbach’s α of the seven-item scale was 0.89.

The fourth section of the questionnaire contained the Copenhagen Psychosocial Questionnaire (COPSOQ). The COPSOQ is designed to assess psychosocial conditions and health promotion in the workplace (Borritz et al., 2006; Burr et al., 2019). It is a generic tool that can be used for all types of workplaces, in all sectors, and for workplaces of different sizes (private or public), and it is a free and public tool. The COPSOQ instrument is standardized with a five-point Likert scale. The scale consisted of different item questions with scores from 1 to 5 for each item, ranging from to a very low degree/never = 1, to a low degree/rarely = 2, to some degree/sometimes = 3, to a high degree/often = 4 and to a very high degree/always = 5, according to their occurrence. As such, the COPSOQ items were grouped into job stress (4 items, *α* = 0.83), depressive symptoms (4 items, *α* = 0.71), sleep problems (4 items, *α* = 0.84), job satisfaction (4 items, *α* = 0.77), role clarity (4 items, *α* = 0.89), recognition (3 items, *α* = 0. 83), job security (2 items, *α* = 0.78), job demands (4 items, *α* = 0.73), student misbehavior (3 items, *α* = 0.83), student demotivation (4 items, *α* = 0.85), social support (3 items, *α* = 0.77) and social relationship (3 items, *α* = 0.83). The COPSOQ is available in several languages (Burr et al., 2019). The COPSOQ is one of the most widely used tools for assessing psychosocial risk factors, and it is cited as a reference in documents of international organizations such as the WHO (Burr et al., 2019) and the International Labor Organization (ILO) (Stress, 2016). It is also recognized as an example of good practice by the European Agency for Safety and Health at Work (Vargas et al., 2014).

### Data quality assurance

To ensure consistency, the questionnaire was first developed in English, then translated into Amharic and back into English by the authors with the help of language experts. We recruited four environmental and occupational health and safety professionals to collect the data and two psychiatric professionals as supervisors with prior experience and knowledge of the data collection process. The data collectors and supervisors received 2 days of training and orientation before the actual data collection on issues such as clarity of questions, the aims of the study, confidentiality of information, and informed consent. The training was given in lecture and discussion formats. The questionnaires were pre-tested on 30 samples that were not included in the final analysis and appropriate modifications were made before the actual data collection. The investigators double-checked the completeness and accuracy of all completed questionnaires daily.

### Data management and statistical analyses

The data were checked for completeness, entered into Epi-data version 4.6, and exported to IBM SPSS 26 software for analysis. Descriptive statistics were presented using, frequency distributions, percentages, means, and standard deviations. Cross-tabulations were used to evaluate the relationship between the outcome variable and the explanatory variables. A binary logistic regression analysis was performed separately for each explanatory variable to explore the associations with the dependent variable (self-reported work-related burnout). Explanatory variables with value of *p*s <0.2 in the bivariate analysis were exported to the multivariable logistic regression model using the backward conditional variable selection method to control for the potential effects of confounders. The Hosmer and Lemeshow goodness-of-fit test was used to check the model fitness (value of *p*s >0.05). The assumption of multi-co-linearity was tested using the variance inflation factor (VIF <5). The adjusted odds ratio (AOR) with 95% confidence intervals (CIs) and a *p*-value <0.05 were applied to establish the significance of associations in the multivariable logistic regression model.

## Results

### Socio-demographic characteristics of participants

A total of 553 out of 588 teachers participated in the study, giving a response rate of 94.05%. The mean age (±SD) of the participants was 42.14 (±7.44) years. Of the total participants, 356 (64.4%) were male and 437 (79%) were married. The majority, 347 (62.8%) had a Bachelor’s degree, and 202 (36.5%) had a Master’s degree. More than half (57.1%) had teaching experience between 11 and 20 years, and 209 (37.8%) of the participants reported teaching for at least 21 years (Table 1).

**Table 1:** Socio-demographic characteristics of public secondary school teachers in Gondar City, Northwest Ethiopia, 2022 (*N* = 553).

Variables	Frequency (*n*)	Percent (%)
Sex
Male	356	64.4
Female	197	35.6
Age in years
23–35	95	17.2
36–45	294	53.2
46–55	126	22.8
≥56	38	6.8
Marital status
Single	116	21
Married	437	79
Level of education
Diploma	4	0.7
Bachelor’s degree	347	62.8
Master’s degree	202	36.5
Teaching experience in years
1–10	28	5.1
11–20	316	57.1
≥21	209	37.8
Daily teaching hours
1–4 h.	458	82.8
5–8 h.	95	17.2
Weekly classes schedules
2–4 days	281	50.8
5–6 days	272	49.2
Monthly salary in ETB
<8,000	13	2.4
8,000–12,579	84	15.2
>12,579	456	82.4
Household members in person
1–4	316	57.1
5–7	223	40.3
>7	14	2.5

ETB, Ethiopian Birr (1$USD = 54.35 ETB currency).

### Behavioral characteristics of participants

Of the study participants, only 75 (13.6%) reported performing physical exercise. Regarding substance use, 177 (32.0%) of the teachers were alcohol drinkers, 12 (2.2%) were cigarette smokers, and 14 (2.5%) were khat chewers (Table 2).

**Table 2:** Behavioral characteristics of public secondary school teachers in Gondar City, Northwest Ethiopia, 2022 (*N* = 553).

Variables	Frequency (*n*)	Percent (%)
BMI
Underweight	31	5.6
Normal	473	85.5
Overweight	49	8.9
Physical exercise
Yes	75	13.6
No	478	86.4
Alcohol drinking
Yes	177	32.0
No	376	68.0
Cigarette smoking
Yes	12	2.2
No	541	97.8
Khat chewing
Yes	14	2.5
No	539	97.5

BMI, Body Mass Index.

### Work-related characteristics of participants

Of the participants, 269 (47.0%) of them were reported that they had high job demand, and 353 (63.8%) did not have job security. Two hundred twenty-seven (41%) of the participants had sleep troubles. Above half, 54.2% of participants were dissatisfied with their job and 254 (45.9%) experienced stress due to their job. The majority, 335 (60.6%) of the participants, were reported to have low social support and just over half (51.7%) were reported to have poor social relationships. Regarding the participants’ conflicts at work, 149 (26.9%) of them reported conflicts with their colleagues and 189 (34.2%) with their school administration, 77 (13.9%) of them with their students. Two hundred and one (36.3%) of the teachers reported that their students misbehaved and 333 (60.2%) of the participants felt that many students lacked motivation for school work. Almost a third (34.2%) of participants planned to leave their job (Table 3).

**Table 3:** Work-related characteristics of public secondary school teachers in Gondar City, Northwest Ethiopia, 2022 (*N* = 553).

Variables	Frequency (*n*)	Percent (%)
Job demand
High	260	47.0
Low	293	53.0
Recognition
No	321	58.0
Yes	232	42.0
Job security
Yes	200	36.2
No	353	63.8
Sleeping troubles
Yes	227	41.0
No	326	59.0
Job satisfaction
Not satisfied	300	54.2
Satisfied	253	45.8
Stress
Stressed	254	45.9
Not stressed	299	54.1
Depressive symptoms
Yes	251	45.4
No	302	54.6
Role clarity
Low	313	56.6
High	240	43.4
Social support
Low	335	60.6
High	218	39.4
Social relation
Poor	286	51.7
Good	267	48.3
Conflict with colleagues
Yes	149	26.9
No	404	73.1
Conflict with management
Yes	189	34.2
No	364	65.8
Conflict with students
Yes	77	13.9
No	476	86.1
Student misbehavior
Yes	201	36.3
No	352	63.7
Student demotivation
Yes	333	60.2
No	220	39.8
Plan to leave the job
Yes	189	34.2
No	364	65.8

### Prevalence of work-related burnout

The results of this study showed that the overall prevalence of work-related burnout (WRB) in the past 12 months among public secondary school teachers was 37.4% (*n* = 207) [95% CI (33.3, 41.6)]. The results of all seven items of work-related burnout and their respective frequency scores and mean scores are presented in Table 4.

**Table 4:** Scales, items and response frequencies of public secondary school teachers in Gondar city, Northwest Ethiopia, 2022 (*N* = 553).

Work-related burnout items (*α* = 0.89)	Response categories and scoring					
	Never (Scoring 0%)	Seldom (Scoring 25%)	Sometimes (Scoring 50%)	Often (Scoring 75%)	Always (Scoring 100%)	Score Mean (SD)
Is your work emotionally exhausting?	9.0	11.8	32.7	30.9	15.6	58.05 (28.61)
Do you feel burnout because of your work?	21.9	19.9	23.1	21.9	13.2	46.16 (33.55)
Does your work frustrate you?	32.0	22.6	20.1	13.9	11.4	37.52 (34.05)
Do you feel worn out at the end of the working day?	4.3	17.4	26.8	29.5	22.1	61.89 (28.51)
Are you exhausted in the morning at the thought of another day at work?	45.8	23.3	17.0	9.80	4.2	25.81 (29.52)
Do you feel that every working hour is tiring for you?	33.3	27.5	17.2	15.2	6.9	33.73 (31.74)
Do you have enough energy for family and friends during your leisure time? (Reversed scoring)	13.2	24.2	34.5	19.9	8.1	46.38 (28.24)
Total average score						45.25 (20.39)

WRB, Work-related burnout.

### Factors associated with work-related burnout

In the bivariate logistic regression analysis, daily teaching hours, job demands, job security, role clarity, social relationship, recognition, job stress, depressive symptoms, job satisfaction, student demotivation, student misbehavior, conflict with colleagues, conflict with management, and conflict with students were factors associated with WRB (value of *p* <0.2). However, after controlling for confounding variables in the final model (multivariable binary logistic regression analysis), job demands, job stress, job satisfaction, role clarity, and student demotivation were found to be significant variables associated with WRB (value of *p* <0.05).

The odds of developing WRB were 2.81 times higher for participants with high job demands compared to those with low job demands [AOR: 2.81; 95% CI (1.83, 4.32)]. Participants who were dissatisfied with their job had about twice the risk of developing WRB compared to those who were satisfied with their job [AOR: 1.94; 95% CI (1.25, 2.99)]. The odds of developing WRB were 3.46 times higher among participants who had experienced job stress than among those who had not [AOR: 3.46; 95% CI (2.14, 5.59)]. Work-related burnout was 1.79 times higher among participants who felt their students were demotivated for schoolwork than among those who did not [AOR: 1.79; 95% CI (1.13, 2.87)]. Participants with low role clarity were 1.84 times more likely to develop WRB than those with high role clarity [AOR: 1.84; 95% CI (1.152, 2.95)] (Table 5).

**Table 5:** Bi-variable and multivariable binary logistic regression analysis of factors associated with work-related burnout among public secondary school teachers in Gondar City, Northwest Ethiopia, 2022 (*N* = 553).

Variables (*N* = 553)	Work-related burnout		COR with 95% CI	AOR with 95% CI
	Yes	No		
Sex
Male	133	223	1	1
Female	74	123	1.01 (0.70, 1.44)	0.89 (0.65, 1.29)
Daily teaching hours
5–8	50	45	**2.13 (1.36, 3.33)**	1.57 (0.91, 2.71)
1–4	157	301	1	1
Job demands
High	135	125	**3.32 (2.31, 4.75)**	2.81 (1.83, 4.32)***
Low	72	221	1	1
Sleep troubles
Yes	118	109	**2.88 (2.02, 4.12)**	1.29 (0.81, 2.08)
No	89	237	1	1
Job stress
Yes	141	113	**4.41 (3.05, 6.37)**	3.46 (2.14, 5.59)***
No	66	233	1	1
Depressive symptoms
Yes	116	135	**1.99 (1.41, 2.83)**	0.87 (0.54, 1.39)
No	91	211	1	1
Role clarity
Low	132	181	**1.60 (1.13, 2.28)**	1.84 (1.15, 2.95)*
High	75	165	1	1
Relationship
Poor	120	166	**1.49 (1.06, 2.12)**	1.19 (0.74, 1.90)
Good	87	180	1	1
Job satisfaction
No	142	158	**2.59 (1.810, 3.733)**	1.94 (1.25, 2.99)*
Yes	65	188	1	1
Student demotivation
Yes	111	109	**2.51 (1.76, 3.59)**	1.79 (1.13, 2.87)*
No	96	237	1	1
Student misbehavior
Yes	96	105	**1.99 (1.39, 2.84)**	0.73 (0.45, 1.19)
No	111	241	1	1
Conflict with colleagues	
Yes	72	77	**1.86 (1.27, 2.73)**	0.57 (0.31, 1.03)
No	135	269	1	1
Conflict with management	
Yes	85	104	**1.62 (1.13, 2.32)**	1.37 (0.79, 2.37)
No	122	242	1	1
Conflict with students	
Yes	38	39	**1.77 (1.09, 2.87)**	1.06 (0.57, 1.99)
No	169	307	1	1

1 = reference category, WRB, Work-related burnout; COR, Crude odds ratio; CI, Confidence interval; AOR, Adjusted odds ratio. *Statistically significant at *p* < 0.05, ***Statistically significant at *p* < 0.0001, Hosmer and Lemeshow test = 0.337. Significant variables in multivariate logistic regression analysis at a *p*-value < 0.05.

## Discussion

Due to the changing nature of modern workplaces, work-related psychosocial hazards are becoming an important area of research. However, little has been documented in developing countries such as Ethiopia, despite its prevalence among workers in different occupations. This study aimed to quantify the prevalence of work-related burnout and its contributing factors among public secondary school teachers in Gondar City, Northwest Ethiopia. This study found that the prevalence of work-related burnout among school teachers in the past 12 months was 37.4% (*n* = 207) [95% CI (33.3, 41.6)]. This implies that more attention should be paid to the well-being of teachers. In Ethiopia, a suboptimal working environment coupled with the traumatic events of daily life conceivably exacerbates the mental health status of workers, including WRB. The prevalence of WRB in this study was comparable to findings from studies in Botswana (40%) (Mogapi and Moorad, 2020), Brazil (36.7%) (Gil-Monte et al., 2011), and Nigeria (36%) (Ozoemena et al., 2021). Possible explanations for this conformity could be that socio-economic status and the nature of work in schools, such as lack of resources (human and material), high workload, job insecurity, and poor recognition and support, are likely to be similar in developing countries.

Our study found a higher prevalence of WRB compared to the studies in Namibia (28.8%) (Louw et al., 2011), the Republic of Srpska (5.1%) (Marić et al., 2020), Morocco (23.9%) (Amri et al., 2021), and Tunisia (27.4%) (Chennoufi et al., 2012). On the other hand, our study found a lower prevalence of WRB compared to studies in Malaysia (50%) (Subon and Sigie, 2016), the USA (52%) (Rumschlag, 2017), and Indonesia (73.3%) (Ramdan et al., 2020). This discrepancy could be due to differences in the assessment tools used, the education systems across the countries, and the timing of data collection. The data in our study were collected at the end of the school year, so teachers may have higher levels of burnout at the end of the school year (Marić et al., 2020). Another possible reason for the difference could be due to teachers’ coping strategies to deal with stressors, cultures of reporting distress, definitions, and levels of perception of WRB across countries (Amri et al., 2021).

The result of our study showed that job demand was significantly associated with WRB. This is consistent with findings from Sweden (Arvidsson et al., 2019), Indonesia (Ramdan et al., 2020), and Morocco (Amri et al., 2021). A possible explanation could be that an increase in high job demands and work tasks may lead to increased time pressure, and less freedom to determine how work is carried out may contribute to increased WRB (Arvidsson et al., 2019). Teachers faced high job demands, including large numbers of students to teach, as well as teaching tasks and ever-changing administrative work, which affected their mental health. They were also at high risk of stress to maintain high school performance. As a result, teaching and non-teaching workloads led to negative reactions, including WRB among teachers (Van Droogenbroeck et al., 2014). The researchers reported that job demands (interpersonal conflicts at work, organizational constraints, quantitative workload) had a stronger effect on mental health (Ibrahim et al., 2021).

There was also a statistically significant relationship between job satisfaction and WRB. This finding is supported by other studies in Malaysia (Henny et al., 2014) and South Africa (Okeke and Mtyuda, 2017). The possible reason could be that job dissatisfaction results from teachers’ high psychological demand and their low control over it (Helms-Lorenz and Maulana, 2016) which then exposes them to frequent psychological loads that increase burnout and other health problems (McCarthy et al., 2015). Although job dissatisfaction can result in discomfort, low self-esteem, sadness, and devaluation, all of which contribute to high levels of stress at work (Wilhelm et al., 2000; Chaplain, 2001) and end up with WRB (Atashpanjeh et al., 2020). The other possible explanation could be that teachers are expected to provide academic instruction, social–emotional support, and build relationships with students and families while often receiving inadequate compensation or support from administration and leadership, which can lead them to dissatisfaction and result in burnout development (Stauffer and Mason, 2013; Nantsupawat et al., 2017; Atashpanjeh et al., 2020). In addition, job dissatisfaction can lead to reduced performance and general well-being, which in turn can lead to WRB (Panagopoulos et al., 2014; Van Droogenbroeck et al., 2014).

In addition, job stress was associated with WRB, which is consistent with previous studies in Germany (Seibt and Kreuzfeld, 2021) and China (Cui et al., 2018) concluding that job stress is a direct cause of WRB. The possible justifications could be that job stress is not only a powerful driving force but also a negative factor influencing work performance and occupational health, creating physical and psychological imbalances. Therefore, high levels of job stress may excessively consume teachers’ emotional and physical resources and ultimately lead them to a severe state of WRB (Veldman et al., 2013).

The results of the current study indicated that teachers with low levels of role clarity in their work were more likely to have WRB than those with high levels of role clarity. This could be because if individuals are uncertain about their responsibilities (if there is little clarity about their job), they may feel stressed and anxious (Blumenthal et al., 1998). This is particularly true in high-pressure work environments such as education, where the stakes are high and the consequences of failure can be severe (Hatcher, 2018). Individuals may feel confused and frustrated if they do not understand what is expected from them. If individuals do not believe they are meeting the expectations of their role, this can lead to feelings of inadequacy. This can be particularly damaging to an individual’s mental health, as it can lead to self-doubt and a lack of confidence in their abilities (Karkkola et al., 2019). Thus, when individuals are uncertain about their roles and responsibilities, they may find themselves working longer hours and taking on more than they can handle. This can lead to WRB and a range of associated mental health problems such as anxiety, depression, and exhaustion (Friesen and Sarros, 1989).

Furthermore, student demotivation was a predictor of WRB in the current study, which is consistent with previous studies conducted in the USA (Aloe et al., 2014) and Morocco (Amri et al., 2021). Student demotivation problems disrupt the teaching-learning process or interfere with the orderly operation of the classroom, so teachers spend a considerable amount of time and energy dealing with student’s disruptive behavior, so this can cause anger and frustration for teachers, these repeated experiences of frustration and anger in response to students lead to WRB (Aloe et al., 2014).

### Strengths and limitation of the study

This study offers great opportunities for educational leaders and other stakeholders to consider the workplace context when planning mental health prevention programs. In Ethiopia, the relationship between various psychosocial work characteristics and mental health symptoms has been little explored, so the current study encourages other researchers to focus on this central issue. However, the results should be interpreted with caution when drawing conclusions. Firstly, the study was a cross-sectional survey limited to educational institutions, i.e., schools, which may make it difficult to extrapolate the results to other workplaces. Secondly, as the data obtained are self-reported past experiences of employees, under-reporting cannot be overlooked. To minimize this bias, we restricted the data to recent experiences only. Future researchers need to include different workplaces to explicitly capture the relationships between different job characteristics and WRB. Despite these limitations, we believe that the study provides an accurate assessment of WRB among public secondary school teachers.

### Implications for practice

The findings of this study have important implications for practice in addressing WRB among public secondary school teachers. The identified contributing factors highlight areas where interventions and strategies can be implemented to support teachers’ well-being and improve their work environment. Implementing stress management programs tailored to the specific needs of teachers to help them cope with work demands and pressures. Promoting job satisfaction through measures such as recognizing, supporting, and providing professional growth opportunities is another effective strategy for managing WRB among teachers. It is also advisable to clarify teachers’ roles and responsibilities to reduce ambiguity and provide a clear sense of purpose. Implementing strategies to increase student motivation and create a positive learning environment is also an area where school leaders need to take action. In addition, fostering a supportive working environment that encourages collaboration, communication, and mutual support among teachers will help to address the psychosocial factors that lead to WRB. By implementing these recommendations, educational authorities, school administrators, and policymakers can work toward creating a supportive and healthy working environment for teachers.

## Conclusion

This study highlights the high prevalence of work-related burnout among public secondary school teachers. Psychosocial work factors such as job demands, job stress, job satisfaction, role clarity, and student demotivation were significant factors influencing work-related burnout. To reduce the condition (WRB), it is recommended to take measures to cope with high job demands, improve stress management skills, promote job satisfaction strategies, clarify teachers’ responsibilities, and use effective teaching practices to motivate students. In general, the management of psychosocial work factors needs to be at the center of efforts to prevent work-related burnout among teachers.

## Data availability statement

The original contributions presented in the study are included in the article/supplementary material, further inquiries can be directed to the corresponding author.

## Ethics statement

The studies involving human participants were reviewed and approved by Psychological Research and the Institute of Public Health, University of Gondar with an approval number of IPH/2107/2014. The patients/participants provided their written informed consent to participate in this study.

## Author contributions

AB, GK, and AT: study conception and design, acquisition of data, analysis and interpretation of data, drafting of the manuscript, and critical revision. KG and GE made substantial revisions to the final version of the manuscript. All authors read and approved the final version of the submitted manuscript.

## Conflict of interest

The authors declare that the research was conducted in the absence of any commercial or financial relationships that could be construed as a potential conflict of interest.

## Publisher’s note

All claims expressed in this article are solely those of the authors and do not necessarily represent those of their affiliated organizations, or those of the publisher, the editors and the reviewers. Any product that may be evaluated in this article, or claim that may be made by its manufacturer, is not guaranteed or endorsed by the publisher.
